# Convolutional MLP orthogonal fusion of multiscale features for visual place recognition

**DOI:** 10.1038/s41598-024-62749-x

**Published:** 2024-05-23

**Authors:** Wenjian Gan, Yang Zhou, Xiaofei Hu, Luying Zhao, Gaoshuang Huang, Chenglong Zhang

**Affiliations:** Institute of Geospatial Information, PLA Strategic Support Force Information Engineering University, Zhengzhou, 450001 China

**Keywords:** Convolutional MLP, Multiscale information, Orthogonal projection decomposition, Enhanced spatial attention, Visual place recognition, Engineering, Computer science, Learning algorithms, Network models

## Abstract

Visual place recognition (VPR) involves obtaining robust image descriptors to cope with differences in camera viewpoints and drastic external environment changes. Utilizing multiscale features improves the robustness of image descriptors; however, existing methods neither exploit the multiscale features generated during feature extraction nor consider the feature redundancy problem when fusing multiscale information when image descriptors are enhanced. We propose a novel encoding strategy—convolutional multilayer perceptron orthogonal fusion of multiscale features (ConvMLP-OFMS)—for VPR. A ConvMLP is used to obtain robust and generalized global image descriptors and the multiscale features generated during feature extraction are used to enhance the global descriptors to cope with changes in the environment and viewpoints. Additionally, an attention mechanism is used to eliminate noise and redundant information. Compared to traditional methods that use tensor splicing for feature fusion, we introduced matrix orthogonal decomposition to eliminate redundant information. Experiments demonstrated that the proposed architecture outperformed NetVLAD, CosPlace, ConvAP, and other methods. On the Pittsburgh and MSLS datasets, which contained significant viewpoint and illumination variations, our method achieved 92.5% and 86.5% Recall@1, respectively. We also achieved good performances—80.6% and 43.2%—on the SPED and NordLand datasets, respectively, which have more extreme illumination and appearance variations.

## Introduction

The process of recognizing and obtaining the geographic location of a given query image in a pre-built image database is known as visual place recognition (VPR), or visual geo-localization (VG). Large-scale image geo-localization is often regarded as an image retrieval task^1,2^. VPR is crucial in many robotics and computer vision tasks, such as autonomous driving^3^, 3D reconstruction^4^, and unmanned aerial vehicle (UAV) localization in Global Navigation Satellite System (GNSS)-denied environments^5^. The challenges of VPR mainly arise from changing external environments, such as different seasons, different illumination, occlusion, and moving objects^6^; environments with high appearance similarities, such as trees and buildings^7^; and differences in camera viewpoints^1^. Therefore, researchers are interested in obtaining feature descriptors that are robust and generalizable to image changes.

Traditional methods use scale-invariant feature transform (SIFT)^8^, and histogram of orientated gradients (HOG)^9^ to obtain local feature descriptors, most current studies^10–14^ insert end-to-end trainable layers into pre-trained feature extraction networks to obtain robust global descriptor representations. Subsequent studies have enhanced the robustness and generalization of image descriptors for better localization by attaching semantic and contextual information^15,16^, using attention mechanisms^10,17^, and exploiting multiscale features^13,18^. Some studies have also achieved more efficient of image retrieval by using a combination of deep features and handcrafted features^19^.

Recent research has concentrated on improving the robustness of image representations by introducing multiscale information in the global image descriptors^20–22^. However, these convolutional neural network (CNN)-based methods either learn multiscale information by constructing image pyramids^18,21^ or extract multiscale features using convolutional kernels of different sizes and dilation coefficients at the last convolutional layer of the model^10,15,20^. These methods ignore the problem of information loss caused by constant downsampling during multiscale feature extraction and the problem of information redundancy when fusing multiscale features.

In this study, we propose a novel feature extraction architecture for the convolutional multilayer perceptron (MLP) orthogonal fusion of multiscale features (ConvMLP-OFMS) to address these problems. While using a convolutional architecture to form a multilayer perceptron to extract more discriminative and robust feature representations, this method makes full use of the scale information generated by feature extraction and eliminates redundant information relative to global descriptors in the scale information through feature projection decomposition. Our approach achieved optimal performance on several VPR benchmark datasets, such as Pittsburgh, Nordland, and MSLS. Our contributions can be summarized as follows:

- We propose a novel MLP-based encoding strategy called ConvMLP, which achieves efficient feature aggregation using a simple architecture and exhibits excellent VPR performance.
- We propose a VPR architecture for orthogonal fusion of multiscale features and demonstrate the effectiveness of multiscale features generated during feature extraction.
- We eliminate noise and redundant scale information by enhancing spatial attention and orthogonal projection decomposition to utilize multiscale information more efficiently.

## Related work

### Visual place recognition

VPR has always been considered an image-retrieval problem^2^, in which the location of a query image is determined according to the known geographical labels of the image in a reference database. Traditional VPR methods use handcrafted feature extraction operators, such as SIFT^8^, and HOG^9^, to obtain the local features of an image. Then, the bag of words (BoW)^23^ and vector of locally aggregated descriptors (VLAD)^24^ are further used to aggregate them into a global descriptor representing the entire image to reduce the computation and storage overhead caused by descriptor dimensions. With the rapid development of computer technology, deep learning methods such as CNN and transformers have achieved excellent results in many computer vision tasks, such as image classification^25,25^ object detection^26^, and image segmentation^27^. Several researchers have used CNNs and vision transformers^28^ to solve VPR problems.

Deep learning-based methods can extract deep semantic information, are more robust to light, weather, and viewpoint changes, and have been widely used in location recognition research^11,15,29^. Arandjelovic et al*.*^11^ proposed NetVLAD, which is a generalized VLAD descriptor that can update parameters through backpropagation. It assigns a feature map to each cluster center pixel-by-pixel using soft assignment, and the cluster centers are continuously updated with training. Another widely used method in VPR is the generalized-mean pooling (GeM)^29^ feature-aggregation method, which is a learnable generalized form of global pooling. The experiments demonstrate that GeM pooling outperforms traditional average and max pooling. Kim et al*.*^15^ introduced a NetVLAD-based contextual reweighting network that assigns different weights to each region of the feature map based on context to obtain richer high-level semantic information and can be combined with other feature-aggregation methods.

The aforementioned methods are all single-stage VPR methods, and are primarily represented by NetVLAD, GeM, and their variants. Similar techniques include the contextual reweighting network (CRN)^15^ and average prevision GeM (AP-GeM)^30^. The main purpose of such single-stage feature coding methods is to obtain more robust, discriminative, and generalized global feature descriptors. Correspondingly, the two-stage rematching methods represented by Patch-NetVLAD^12^, TransVPR^17^, and R2Former^31^; primarily optimize the retrieval results of global features by using local features to achieve the purpose of improving the retrieval accuracy.

**Figure 1:**
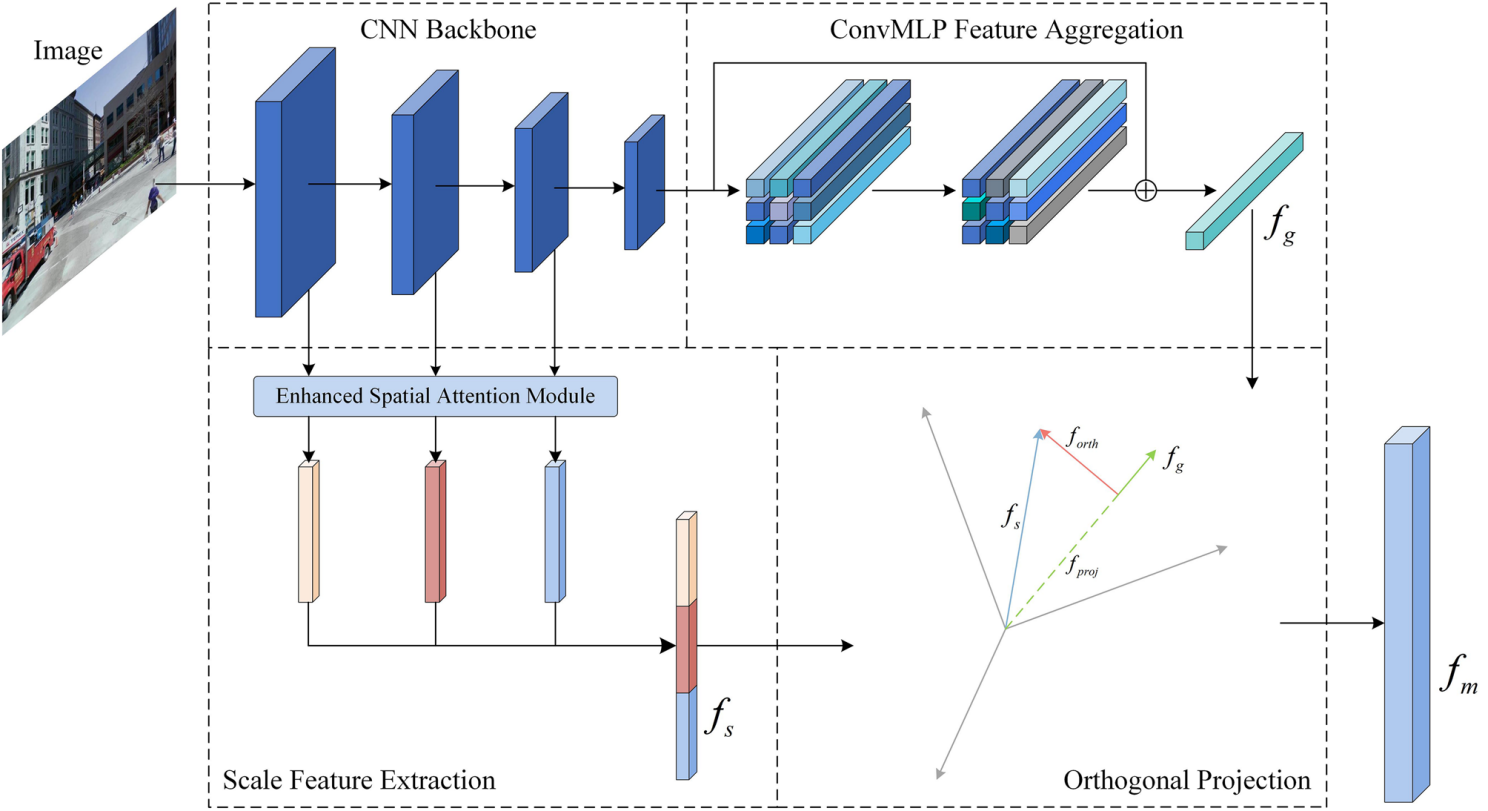
Overview of the proposed architecture for visual place recognition (VPR).

Despite the effectiveness of these end-to-end VPR methods, the acquisition of robust and accurate global image descriptors requires continuous attention, owing to the complexity and variability of the external environment in practical applications. Therefore, we propose a novel single-stage VPR architecture, as shown in Fig. 1. First, ResNet50 is used as the backbone for feature extraction. Then, ConvMLP is proposed for feature aggregation to obtain a robust global descriptor, and enhanced spatial attention (ESA) is used to remove the noise from the multiscale information. Finally, projection decomposition is performed on the multiscale information to remove redundant information that already exists in the global descriptor. The proposed method can also be applied to other computer vision tasks such as human activity recognition^32^ and person reidentification^33^.

### MLP-based methods

Deep learning-based approaches to computer vision have been dominated by CNNs and vision transformers^34^. However, in recent years, architectures based entirely on MLPs have achieved similar performances to CNNs and transformers in image classification. Tolstikhin et al*.*^35^ proposed an MLP-mixer that uses token- and channel-mixed MLPs to allow interaction between the spatial location and channel information. Touvron et al*.*^36^, inspired by ResNet^25^ and the Vision Transformer^28^, designed ResMLP, which allows long-distance interaction of feature information for image classification. Liu et al*.*^37^ proposed an MLP-based transformer alternative (gMLP) consisting only of channel projections with static parameterization and spatial projections by adding spatial gating units to connect channel MLPs and interact with spatial information. Fekri et al*.*^38^ proposed an image classification method that improves accuracy by utilizing MLPs to unify the advantages of CNNs with different architectures. These MLP-based model demonstrated that a simple feedforward neural network can achieve performance similar to that of convolutional operations and self-attention in image classification.

Encoding spatial information using an MLP requires that the input dimensions be fixed. However, the resolution of the input image is typically not fixed. Although this problem can be solved by resizing the input image, this method inevitably results in the loss of image information, which affects the final retrieval and localization results.

In this study, owing to the similarities between VPR and image classification^39^, we propose a novel and efficient feature-aggregation technique called ConvMLP based on the aforementioned MLP-related studies. We used a 1 × 1 convolution to form an MLP that highlights the channel information and adaptive average pooling to strengthen the spatial information in obtaining global features while avoiding information loss due to image resizing.

### Leveraging multiscale information

Several studies have shown that multiscale information can be utilized to effectively improve performance of a model in the VPR task^10,13,18,29,40,41^; this approach can effectively avoid the problem of localization failure caused by the scale difference between the query image and the reference image. The differences between these approaches are how the multiscale information is obtained and how to collect more spatial contextual information when considering multiscale information.

Yu et al*.*^13^ used pyramid pooling comprising convolutional kernels of different sizes to augment the vectors of locally aggregated descriptors, thereby allowing the augmented VLAD vectors to reflect the spatial layout information of the image. Khaliq et al*.*^18^ augmented NetVLAD using low-resolution images at different scales, which resulted in a richer representation of the location. Radenovic et al*.*^29^ used multiple image resolutions for training and then aggregated all the features from different resolutions to get more robust global features.

Zhu et al*.*^10^ used attention-enhanced spatial pyramid pooling for feature encoding, which distinguished regional features while confusing them. Peng et al*.*^40^ used a semantically enhanced local weighting scheme for local feature refinement and then constructed an attention pyramid based on the spatial saliency of regional features for the adaptive encoding of local features. Xu et al*.*^41^ used an attention-based sparse encoder to obtain feature maps, thereby capturing global dependencies. Then, self-supervised learning was utilized to further acquire multiscale information between query images, effectively reducing the visual ambiguities arising in large-scale VPR. Kushwaha et al*.*^42^ used convolutional kernels of different sizes to obtain multi-scale information to address the problem of category differences in complex patterns.

Current methods either use a multi-resolution image pyramid for training or use convolutional kernels with different receptive fields to capture multiscale semantic information on the feature map acquired by the last CNN layer of the model. These methods do not consider the problem of information loss due to constant down-sampling during feature extraction, nor do they fully utilize the multiscale features generated during feature extraction. Thus, in this study, we used the multiscale information generated during feature extraction for multiscale feature fusion, which avoids the problem of large computations caused by using dilated convolution in the deep layer of the network. For the feature redundancy problem that exists in traditional feature fusion methods, spatial attention and orthogonal projection of features are used to eliminate redundant information in multiscale features as much as possible, and the experiments prove the effectiveness of the proposed method.

## Methodology

In this section, we first introduce the proposed ConvMLP aggregation method, then describe how we obtain the multiscale features and the feature orthogonal fusion method used. Finally, we briefly introduce the loss function used by our architecture.

### Convolutional MLP for feature aggregation

Based on a related study of MLPs, we propose a fully convolutional MLP architecture called ConvMLP, which can be used to learn robust feature descriptors. For a given input image $I \in R^{{3 \times h_{0} \times w_{0} }}$, we refer to the approach used by Berton et al*.*^43^ and Ali-Bey et al.^44^ and use ResNet50, which is pretrained on ImageNet by cropping off the last convolutional and classification layers, as a feature extraction method to obtain a tensor $F \in R^{c \times h \times w}$, where $h \times w$ is the feature map size and $c$ is the number of feature map channels.

A set of 1 × 1 convolutions was used to form a multilayer perceptron, and for the feature map $F \in R^{c \times h \times w}$ acquired by the backbone network, feature aggregation was achieved by stacking multiple layers of the ConvMLP. The expression for ConvMLP is as follows:

1 $$\begin{array}{*{20}c} {ConvMLP\left( F \right) = F + W_{2} \left( {\sigma \left( {BN\left( {W_{1} \left( F \right)} \right)} \right)} \right)} \\ \end{array}$$

where *W*_1_ and *W*_2_ represent the 1 × 1 convolution, $\sigma$ is the ReLU nonlinear activation, and *BN* represents batch normalization; inductive bias was not used.

**Figure 2:**
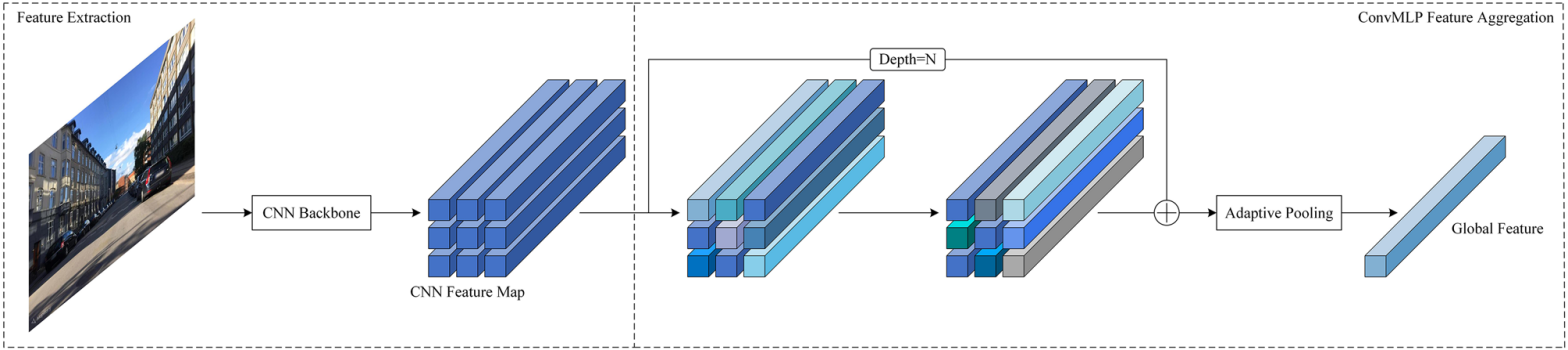
Schematic of the ConvMLP structure.

Thereafter, the spatial dimension of the feature map was transformed from $h \times w$ to 1 × 1 using adaptive average pooling. Finally, the dimensionality-decreased feature map was flattened and $L_{2}$ normalized to obtain the global descriptor $f_{g} \in R^{c \times 1}$ used to represent the entire image. In this study, the actual dimension of $f_{g}$ was 1024 since the feature map from ResNet50 is used as input. Figure 2 illustrates the basic flow. The proposed method can be expressed as follows:

2 $$\begin{array}{*{20}c} {f_{g} = AAP\left( {ConvMLP_{D} \left( {ConvMLP_{D - 1} \left( { \cdot \cdot \cdot ConvMLP_{1} \left( F \right)} \right)} \right)} \right)} \\ \end{array}$$

where $AAP$ represents adaptive average pooling, $ConvMLP$ represents the convolutional multilayer perceptron, $F \in R^{c \times h \times w}$ represents the feature map obtained using ResNet50, and $D$ represents the depth of the ConvMLP.

In summary, instead of focusing on local features or using the attention mechanism, the 1 × 1 convolutional property was used to aggregate channel features, and adaptive mean pooling was used to aggregate spatial features. Based on MLP research, a feature-aggregation method called ConvMLP is proposed, which attempts to avoid excessive parameter count and computation while fully performing feature aggregation.

### Multiscale feature orthogonal fusion

A common approach used in VPR is spatial pyramid pooling or similar structures to obtain spatial features at different scales. However, as stated in section “Leveraging multiscale information”, this method is only applied to the feature map extracted from the last convolutional layer of the model and does not consider the multiscale information generated during feature extraction; therefore, we use the features at different scales generated during feature extraction for feature fusion.

**Figure 3:**
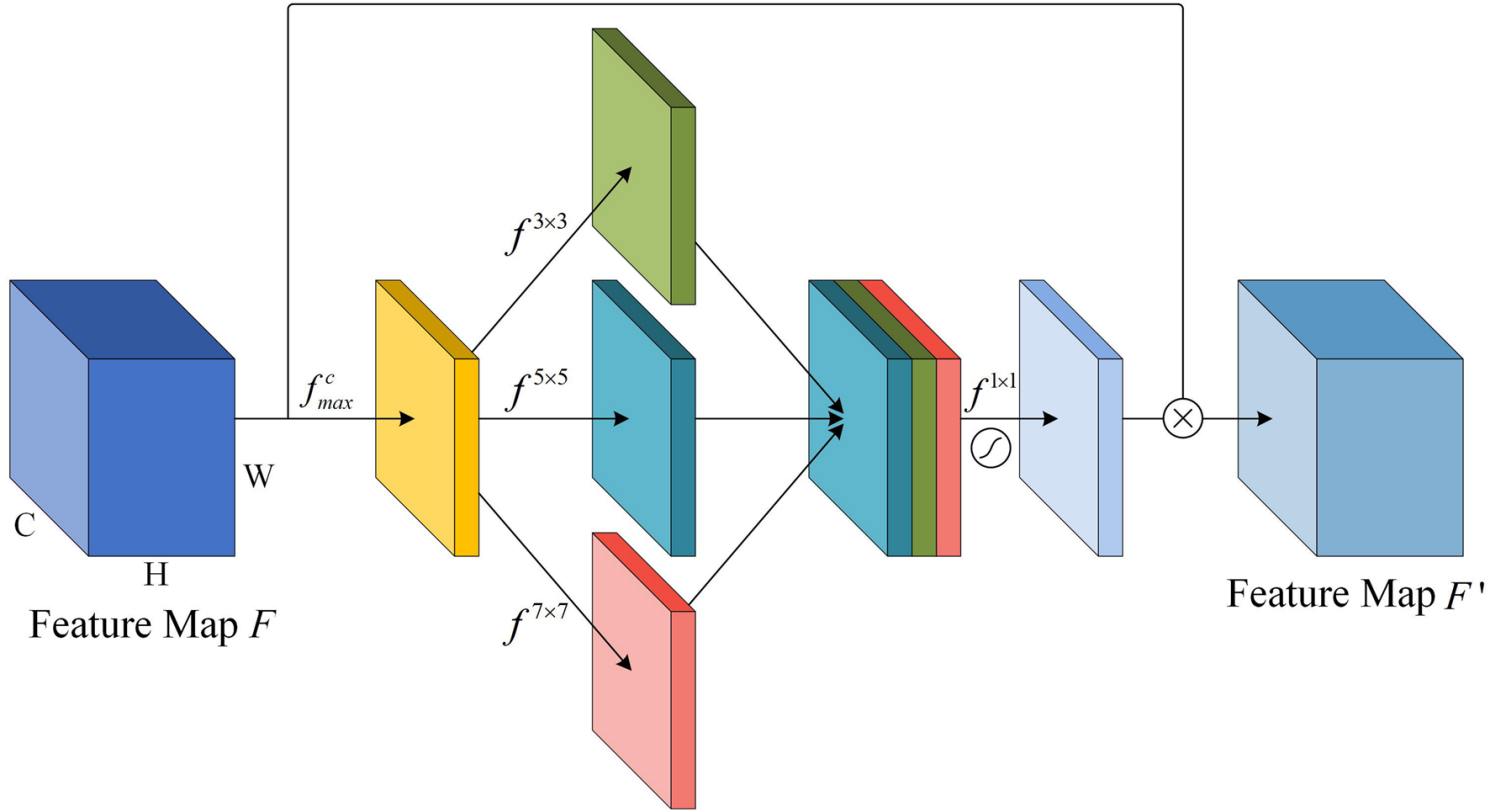
ESA module.

Considering that feature extraction often produces shallow features that contain more noise, we propose an ESA module based on the method propose by Woo et al*.*^45^, as shown in Fig. 3. This module can embed spatial layout information into the feature representation, such that the network focuses on regions that are valuable for VPR and suppresses other irrelevant objects and noise.

The ESA module can be represented as follows:

3 $$\begin{aligned} S\left( F \right) & = f_{max}^{c} \left( F \right) \\ M\left( F \right) & = \delta \left( {f^{1 \times 1} \left( {f^{3 \times 3} \left( {S\left( F \right)} \right) \cup f^{5 \times 5} \left( {S\left( F \right)} \right) \cup f^{7 \times 7} \left( {S\left( F \right)} \right)} \right)} \right) \\ F^{\prime} & = M\left( F \right) \otimes F \\ \end{aligned}$$

For a feature map $F \in R^{c \times h \times w}$, we first used max pooling along the channel to highlight the more valuable features. Thereafter, convolution kernels with different receptive fields were used to focus on more scale information. The three feature maps containing different scales were spliced along the channel direction, and the attention scores of the input features were obtained using a 1 × 1 convolution and sigmoid function. Finally, the attention scores were broadcast onto the input feature map to obtain the spatial attention-weighted features. The feature maps obtained from the first three convolutional stages of ResNet50 were spatially attention-weighted and then fused in the channel direction to obtain multiscale features $f_{s} \in R^{c \times h \times w}$. To facilitate the orthogonal decomposition in the next step, we performed a dimensional transformation of the feature map obtained from the first convolutional layer of ResNet50 using a normal convolution, thus keeping the dimensions of $f_{s}$ and $f_{g}$ equal. Thus, the channel dimension of $f_{s}$ was also 1024, but the spatial information was preserved, and its actual dimensions were 1024 × *h* × *w*.

Traditional multiscale feature fusion is usually realized by tensor splicing; however, this method does not consider the repetition and redundancy between different descriptors, which will have an impact on retrieval accuracy. Therefore, we adopted a method of feature orthogonal projection fusion^46,47^, by which orthogonal projection decomposition can eliminate redundant scale information. Thus, the scale and global information obtained can be enhanced to generate more compact image descriptors.

**Figure 4:**
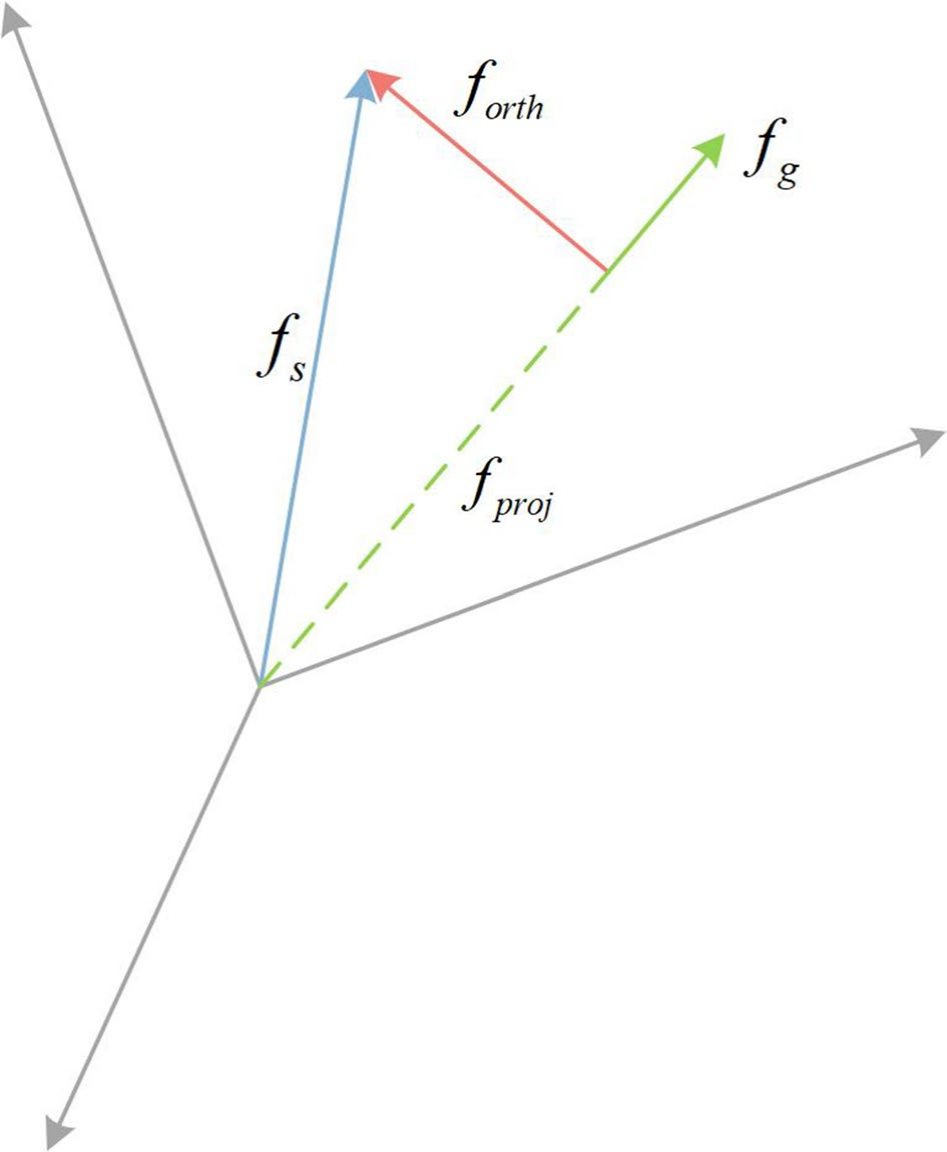
Demonstration of the projection of a multiscale feature onto a global feature.

The basic principle of feature orthogonal projection fusion is shown in Fig. 4. It requires the global feature $f_{g}$ and multiscale feature $f_{s}$ as inputs and calculates the projection $f_{s,proj}^{{\left( {i,j} \right)}}$ of each multiscale feature $f_{s}^{{\left( {i,j} \right)}}$ on the global feature $f_{g}$ pixel-by-pixel, as follows:

4 $$\begin{array}{*{20}c} {f_{s,proj}^{{\left( {i,j} \right)}} = \frac{{f_{s}^{{\left( {i,j} \right)}} \cdot f_{g} }}{{\left| {f_{g} } \right|^{2} }}f_{g} } \\ \end{array}$$

The corresponding orthogonal component $f_{s,orth}^{{\left( {i,j} \right)}}$ was then obtained by computing the difference between the multiscale feature $f_{s}^{{\left( {i,j} \right)}}$ and its projection vector $f_{s,proj}^{{\left( {i,j} \right)}}$, as follows:

5 $$\begin{array}{*{20}c} {f_{s,orth}^{{\left( {i,j} \right)}} = f_{s}^{{\left( {i,j} \right)}} - f_{s,proj}^{{\left( {i,j} \right)}} } \\ \end{array}$$

Next, for the orthogonal component $f_{s,orth}^{{\left( {i,j} \right)}} \in R^{c \times h \times w}$, following the previous method for obtaining global features, the feature map of the orthogonal components was aggregated into $c \times 1$ orthogonal descriptors $f_{orth}$ using adaptive average pooling and $L_{2}$ regularization. Finally, $f_{orth}$ and $f_{g}$, which had identical dimensions, were concatenated in the channel dimension to obtain the mixed descriptor $f_{m}$ for image retrieval localization.

### Multi-similarity loss

Existing VPR methods^11,13,18^ typically use triplet loss^48^ as the loss function of the model, which achieves weakly supervised training by mining the intraclass positive samples $p$ corresponding to the anchor samples $a$ and interclass negative samples $n$. For VPR, $a$ is typically a single-query image, and $p$ and $n$ are typically determined from the ground truth of the images in the reference image database.

6 $$\begin{aligned} {\mathscr{L}}_{{triplet{ - }loss}} & = \mathop \sum \limits_{i}^{N} \left[ {\left\| {f\left( {x_{i}^{a} } \right) - f\left( {x_{i}^{p} } \right)} \right\|_{2}^{2} - \left\| {f\left( {x_{i}^{a} } \right) - f\left( {x_{i}^{n} } \right)} \right\|_{2}^{2} + \alpha } \right]_{ + } \\ & = \max \left( {D\left( {a,p} \right) - D\left( {a,n} \right) + \alpha ,0} \right) \\ \end{aligned}$$

Triplet loss uses the Euclidean distance as a metric; + indicates that the value in parentheses is taken as the loss value when the value is greater than zero, and the loss value is taken as zero when it is less than zero. $\alpha$ is a threshold set to help the model learn, its value is empirically determined and is usually set to 0.1. To improve the generalization ability of the model, it is common to select positive samples $p$ with negative samples $n$, such that $D\left( {a,n} \right) < D\left( {a,p} \right)$, which is also known as the hard negative-sample selection strategy.

We chose the multi-similarity loss function^49^ for training, which has been shown to exhibit the best performance in VPR. Multi-similarity loss mitigates the problem of excessively large interclass distances and excessively small intraclass distances in metric learning by considering multiple similarities. Instead of using absolute spatial distances as the only metric, it uses the overall distance distribution of the other pairs of samples in the batch to weight the loss, as follows:

7 $$\begin{array}{*{20}c} {{\mathscr{L}}_{MS} = \frac{1}{N}\mathop \sum \limits_{i = 1}^{N} \left\{ {\frac{1}{\alpha }\log \left[ {1 + \mathop \sum \limits_{{j \in {\mathcal{P}}_{i} }} e^{{ - \alpha \left( {S_{ij} - m} \right)}} } \right] + \frac{1}{\beta }\log \left[ {1 + \mathop \sum \limits_{{k \in {\mathcal{N}}_{i} }} e^{{\beta \left( {S_{ik} - m} \right)}} } \right]} \right\}} \\ \end{array}$$

where ${\mathcal{P}}_{j}$ represents the set of positive sample pairs for each instance in each batch; ${\mathcal{N}}_{i}$ represents the set of negative-sample pairs for each instance in each batch; $S_{ij}$ and $S_{ik}$ denote the similarities between the two images; and $\alpha$, $\beta$, and $m$ are fixed hyperparameters.

## Experiential results

In this section, we validate the proposed method on several VPR benchmark datasets and compare it with several state-of-the-art VPR methods to demonstrate the superiority of the proposed method. We describe some of the details of the experiments, including the hyperparameters used, datasets, and evaluation metric (section “Implementation details”). We show the comparison and analysis of our proposed ConvMLP orthogonal fusion of multiscale features method with several other VPR methods in our experimental results (section “Comparison with existing methods”). We demonstrated the effectiveness of each component used in our architecture through ablation experiments (section “Ablation studies”).

### Implementation details

#### Parameters

We used ResNet50, pre-trained on ImageNet with the last convolutional and classification layers trimmed off, as the feature extraction backbone and trained it on the GSV-Cities^50^ dataset, which is a large-scale dataset consisting of more than 560,000 images containing more than 67,000 places. As in the approach used by Ali-Bey et al*.*^50^, we used multi-similarity loss as the loss function of the model, where each batch contained 120 locations, and 4 images were randomly selected for each location; thus, the batch size was 120 × 4 = 480. We used SGD for optimization, with an initial learning rate of 0.05, momentum of 0.9, and weight decay of 0.001; additionally, we used MultiStepLR to decrease the learning rate to the original 0.3 every five epochs. We trained for a maximum of 30 epochs using images that were resized to 320 × 320 pixels.

#### Datasets

We used four datasets—Pittsburgh^7^, MSLS^51^, SPED^52^, and Nordland^52^—to evaluate the proposed architecture. We used two subsets of Pittsburgh: Pitts250k, which contained 8280 query images and 83,952 reference images, and Pitts30k, which contained 7608 query images and 10,000 reference images, all of which were collected from Google Street View and were mainly characterized as viewpoint changes. The MSLS contained 11,120 query images and 18,916 reference images collected from a dashcam, which contained significant viewpoints and lighting variations. The SPED contained 607 query images and 607 reference images collected from surveillance cameras, which primarily represent intense illumination changes and seasonal variations. Nordland contained 2760 query images and 27,592 reference images, including extreme illumination and appearance changes, making it a challenging dataset.

#### Evaluation metric

We followed the same evaluation metrics as in previous studies^11,18,43^ and used Recall@N as a metric to evaluate the model capability. A query image was considered to be successfully retrieved if at least one of the first N retrieved reference images was located within 25 m of the query image.

### Comparison with existing methods

#### Comparing with single-stage framework

In this section, we compare several single-stage VPR methods based on the global descriptors, AVG^11^, GeM^29^, NetVLAD^11^, SPE-NetVLAD^13^, GatedNetVLAD^53^, CosPlace^39^, and ConvAP^50^, with the proposed ConvMLP-OFMS architecture. All methods used the same feature extraction network and were trained on GSV-Cities. We also referred to some of the works in^50^ and the final results are listed in Table 1.

**Table 1:** Comparison of different techniques for popular benchmarks. The baseline represents the global feature descriptor obtained using adaptive average pooling. Significant values are in bold.

Method	FLOPs (GF)	Params (MB)	Latency (ms)	Pitts250k			MSLS			SPED			Nordland		
				R@1	R@5	R@10	R@1	R@5	R@10	R@1	R@5	R@10	R@1	R@5	R@10
AVG^11^	6.78	8.54	4.87	78.3	89.8	92.6	73.5	83.9	85.8	58.8	77.3	82.7	15.3	27.4	33.9
GeM^29^	8.43	23.51	4.99	82.9	92.1	94.3	76.5	85.7	88.2	64.6	79.4	83.5	20.8	33.3	40.0
NetVLAD^11^	6.80	8.61	6.86	90.5	96.2	97.4	82.6	89.6	92.0	78.7	88.3	91.4	32.6	47.7	53.3
SPE-NetVLAD^13^	20.65	44.27	8.49	89.2	95.3	97.0	78.2	86.8	88.8	73.1	85.5	88.7	25.5	40.1	46.1
Gated NetVLAD^53^	–	–	–	89.7	95.9	97.1	82.0	88.9	91.4	75.6	87.1	90.8	34.4	50.4	57.7
CosPlace^39^	8.44	27.70	4.87	91.5	96.9	97.9	84.5	90.1	91.8	75.3	85.9	88.6	34.4	49.9	56.5
ConvAP_2×2_^50^	7.20	9.59	4.72	92.4	97.4	98.4	83.4	90.5	92.3	80.1	90.3	93.6	38.2	54.8	61.2
Baseline	6.78	8.54	4.41	77.8	89.0	91.9	71.5	81.6	85.7	61.9	79.4	83.0	17.6	29.6	37.5
ConvMLP	7.62	10.64	4.55	91.6	97.1	98.2	84.1	90.2	92.3	77.6	89.6	91.9	42.6	58.8	64.9
ConvMLP-OFMS	10.45	17.74	5.13	**92.5**	**97.4**	**98.6**	**86.5**	**92.3**	**93.8**	**80.6**	**90.6**	**93.6**	**43.2**	**60.3**	**66.3**

Bold indicates optimal results.

**Figure 5:**
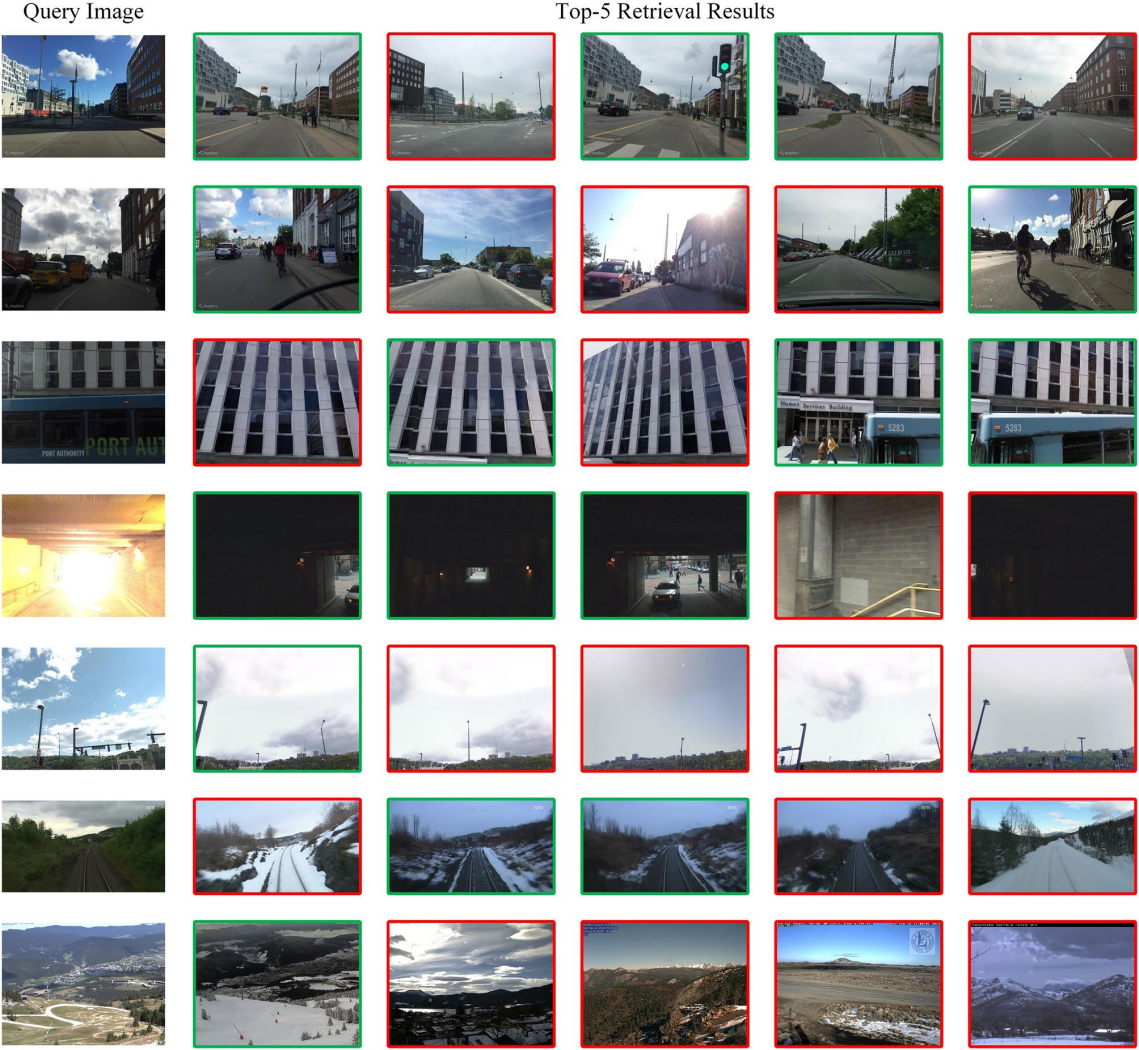
Top five results of our method retrieval; green indicates correct retrieval results.

As shown in Table 1, the proposed method outperforms other methods on several VPR benchmark datasets. On the Pitts250k dataset, we achieved 92.5% Recall@1, which is a slight improvement over the previous methods. On the MSLS dataset, we obtained 86.5% Recall@1, representing improvements of 2% and 3.1% compared with CosPlace and ConvAP, respectively. This demonstrates the ability of the proposed architecture to cope effectively with viewpoint and illumination variations in the VPR. On the SPED and NordLand datasets, which have extreme illumination and appearance variations, we achieved optimal performances of 80.6% and 43.2%, respectively. In addition, as shown in Table 1, after the orthogonal fusion of multiscale features based on ConvMLP, Recall@1 is improved on all datasets by up to 3%, indicating the effectiveness of the orthogonal fusion of the multiscale information encoding strategy that we have adopted. Figure 5 shows the top five retrieval results of our method under difficult conditions, and it can be seen that our proposed method is successful in localization even in extreme environmental changes.

We also compare the computational cost of our proposed method in terms of the floating point operations (FLOPs), number of parameters, and inference time for a single image; the results are shown in Table 1. Our method has a higher computational cost than the AVG and NetVLAD methods, but is far superior in terms of Recall@1. Although the FLOPs and parameter counts of our proposed ConvMLP are slightly higher than those of ConvAP, the inference is faster, the recall performance is superior, and the comprehensive performance is better. After orthogonal fusion of multiscale features on top of ConvMLP, the recall performance is improved, despite the increased computational cost. The performance improvement is particularly clear on the MSLS and SPED datasets. Moreover, compared to other methods that utilize multiscale information, our method sacrifices less for computational cost and performs better, which fully demonstrates the superiority of our proposed method.

#### Comparing against two-stage methods

As mentioned in section “Visual place recognition”, our proposed method belongs to the single-stage framework, and there is another class of methods belonging to the two-stage framework, which primarily uses local features to optimize the retrieval results of the single-stage framework. It is well known that this can significantly improve performance, but at the cost of more computation time and memory. We compared it with SuperGlue^54^, Patch-NetVLAD^12^, TransVPR^17^, and R2Former^31^, all of which are advanced two-stage techniques. Table 2 shows the results of the comparison with two-stage techniques, from which it can be seen that our method outperforms most two-stage techniques in terms of Recall@N performance, and at the same time, significantly outperforms existing two-stage methods in terms of the latency time. Although our method does not perform reranking, the performance of our method is worse than that of the existing state-of-the-art R2Former on the MSLS dataset, and even outperforms R2Former on the Pitts30k dataset, with an improvement of 0.6% in Recall@1 and 1.8% in Recall@5. Meanwhile, our method takes only 5.1 ms to complete the feature extraction of an image, which is faster than all existing methods, and does not require equivalent time for reranking.

**Table 2:** Comparing against two-stage methods in Recall@N, Extraction and Reranking Latency per query is measured on MSLS using NVIDIA RTX A5000. Reranking is done for the top 100 candidates. Significant values are in bold.

Method	Pitts30k			MSLS			Latency per query (ms) ↓	
	R@1	R@5	R@10	R@1	R@5	R@10	Extraction	Reranking
SuperGlue^54^	87.2	94.8	96.4	78.1	81.9	94.3	6.9	3041.2
Patch-NetVLAD^12^	88.7	94.5	95.9	79.5	86.2	97.7	9.3	8377.7
TransVPR^17^	89.9	94.9	96.2	86.8	91.2	92.4	6.2	1757.7
R2Former^31^	91.1	95.2	96.3	**89.7**	**95.5**	**96.2**	8.1	202.4
Ours	**91.7**	**97.4**	**98.5**	86.5	92.3	93.8	**5.1**	0

Bold indicates optimal results.

#### Comparison with methods utilizing multiscale information

**Figure 6:**
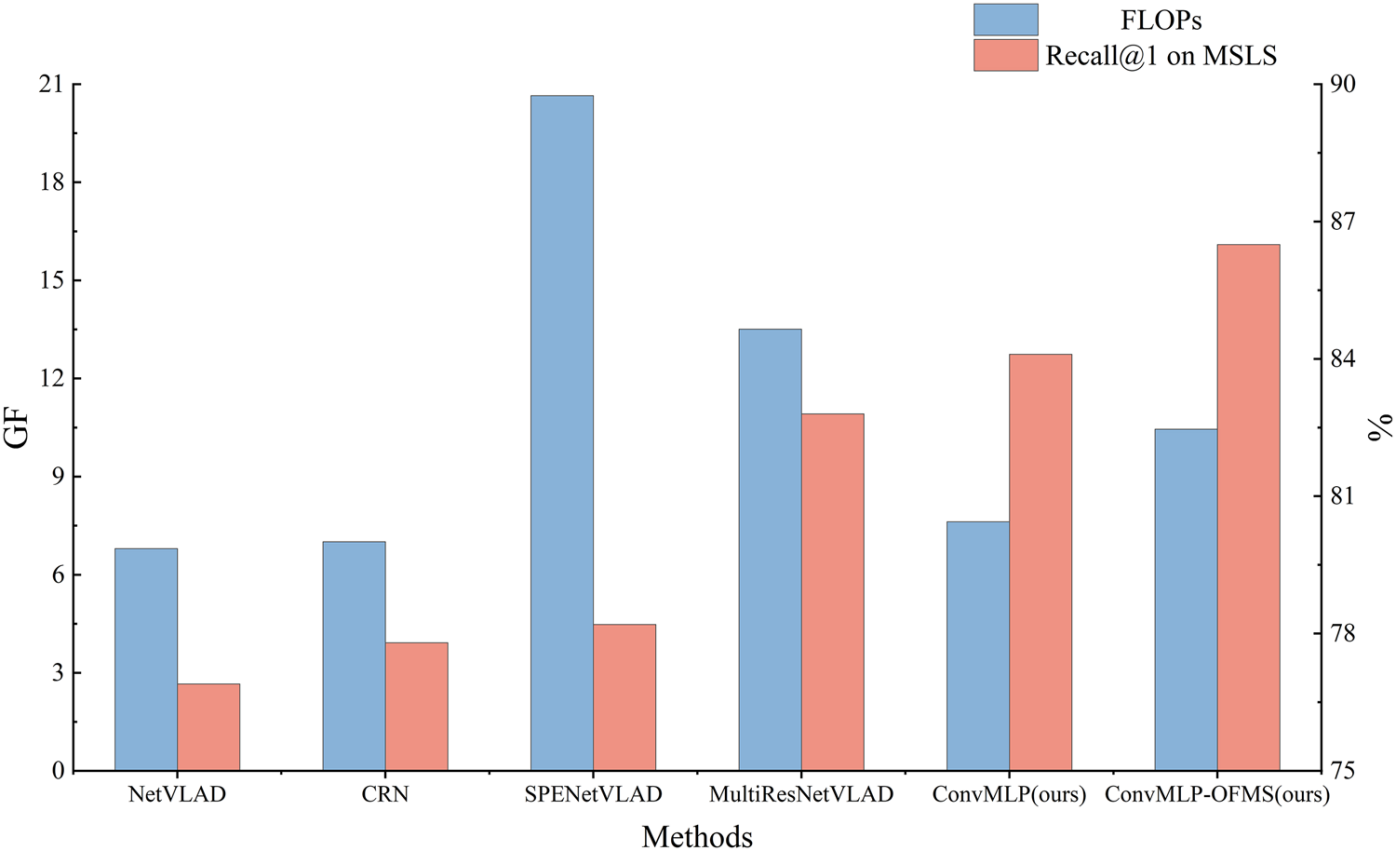
Comparison of FLOPs and Recall@1 on MSLS.

As shown in Fig. 6, compared to other VPR methods utilizing multiscale information, our proposed architecture not only has a high retrieval precision with 86.5% Recall@1 on the MSLS dataset, which is 8.3% and 3.7% higher than SPE-NetVLAD^13^ and MultiRes-NetVLAD^18^, respectively, but also requires only 50.6% and 77.4% as many FLOPs compared to the former and the latter.

### Ablation studies

#### Importance of ConvMLP

To reflect the role of ConvMLP, the global descriptors for retrieval in an ablation experiment were obtained in this section by stacking the number of ConvMLPs $D$. Table 3 presents the results. We set $D \in \left\{ {0,\;1,\;2,\;4} \right\}$ to perform four sets of experiments; when $D = 0$, which is the baseline model we used, the global descriptors were obtained using adaptive average pooling for the feature maps obtained from the backbone network. When $D = 1$, the Recall@1 performance for Pitts30k increased from 83.94 to 91.67%, an improvement of 7.73%, and the performance for MSLS increased from 71.49 to 84.05%, an improvement of 12.56%. Further increases in D produced little improvement in the results for the Pitts30k and MSLS datasets and the accuracy deteriorated. Considering the increase in the number of parameters and FLOPs caused by stacking the ConvMLP, we chose to use $D = 1$ as the benchmark.

**Table 3:** Ablation of ConvMLP blocks. Significant values are in bold.

× *D*	Params (MB)	FLOPs (GF)	Pitts30k			MSLS		
			R@1	R@5	R@10	R@1	R@5	R@10
0	8.54	6.78	83.94	93.94	96.03	71.49	81.62	85.68
1	10.64	7.62	91.67	**97.34**	**98.33**	**84.05**	90.24	**92.30**
2	12.74	8.46	**91.75**	97.31	98.28	83.65	**90.49**	92.03
4	16.94	10.14	91.11	97.31	98.26	83.92	90.41	92.16

Bold indicates optimal results.

**Figure 7:**
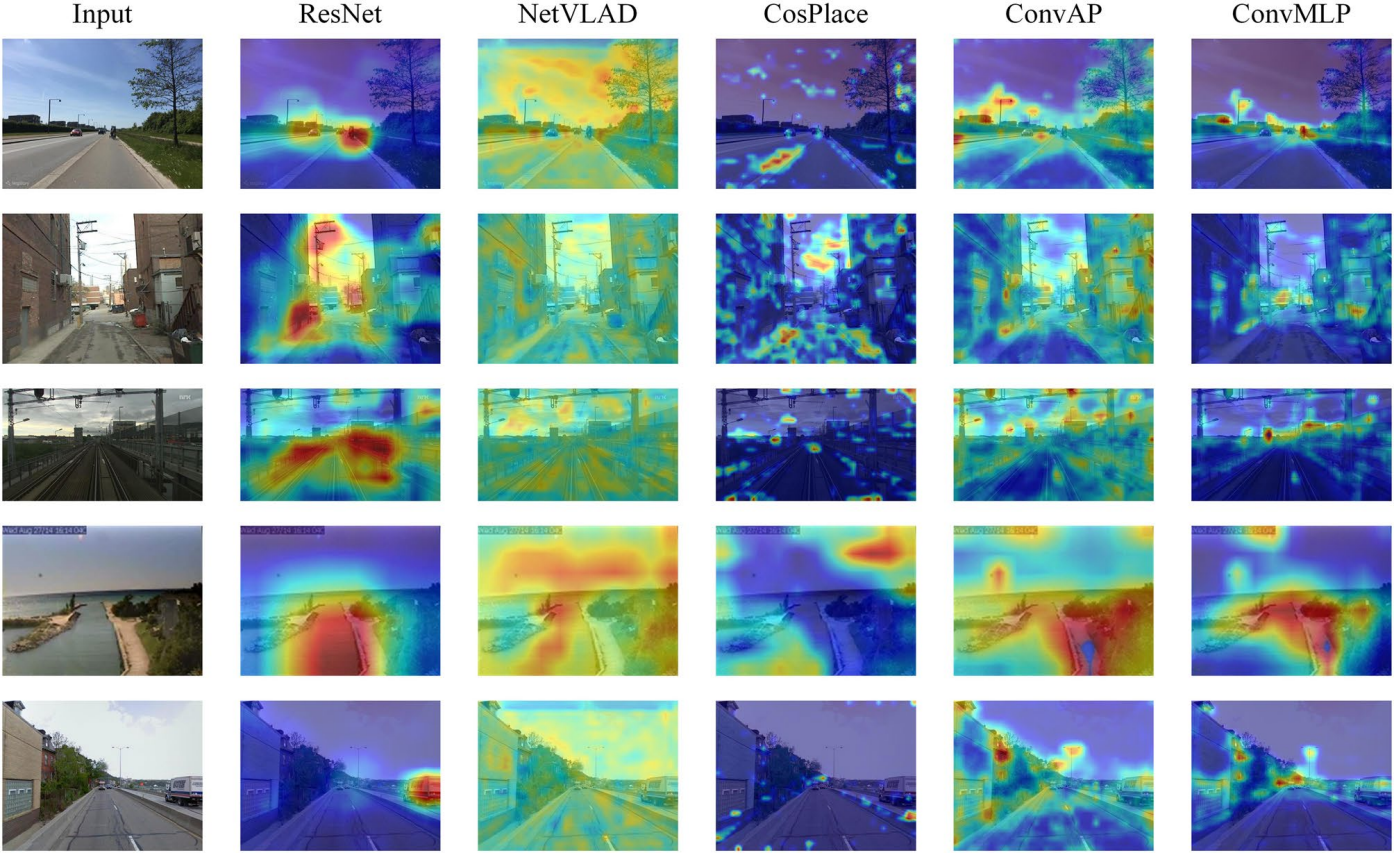
Heat maps of input image feature extraction using different methods.

To illustrate the feature extraction and expression ability of the ConvMLP more intuitively, the heat maps of several methods on the input images are presented in Fig. 7, and the darker color indicates that the model pays more attention to the region. Compared with the ResNet50, CosPlace, and ConvAP methods, the proposed ConvMLP can more accurately highlight the content of the query image, which indicates that the method has a stronger ability to express the key features and can efficiently extract the more critical semantic feature information in the query image, thus achieving better performance.

#### Effects of enhanced spatial attention

This section demonstrates the effectiveness of the proposed ESA method through experimental comparisons. The experimental results are listed in Table 4. The results show that adding the ESA improves Recall@1 on the Pitts30k and MSLS datasets by 1.6% and 3.65%, respectively. It also indicates that adding the ESA can effectively remove the noise of the shallow network and make the model focus on more valuable information for VPR.

**Table 4:** Effectiveness of ESA. “Multiscale” denotes the retrieval query using only the multiscale features $f_{s}$ generated during the feature extraction, and MS + ESA denotes the experimental results of adding ESA to the multiscale features. Significant values are in bold.

Method	Pitts30k			MSLS		
	R@1	R@5	R@10	R@1	R@5	R@10
Multiscale	81.76	93.89	96.19	66.62	78.38	81.62
MS + ESA	**83.36**	**94.42**	**96.37**	**70.27**	**80.41**	**83.92**

Bold indicates optimal results.

This experiment also shows that if the multiscale information generated in the feature extraction is used alone to construct image descriptors for retrieval and recognition, the Recall@1 on Pitts30k and MSLS is 8.31% and 13.78% lower than those of the global image descriptors obtained by ConvMLP, respectively. This indicates that the construction of image descriptors using the multiscale information generated during feature extraction alone is not applicable to solving the VPR problems because the multiscale information generated during feature extraction contains more shallow features, and the deep semantic information is under-represented. Combined with the experimental results in Table 1, it illustrates that using multiscale information to enhance the global descriptors obtained by ConvMLP can effectively increase the robustness and generalization of the descriptors, which again proves the effectiveness of the adopted orthogonal fusion of the multiscale feature strategy.

#### Validation of the orthogonal fusion module

To demonstrate the effectiveness of the orthogonal fusion, we conducted a comparison experiment by removing the orthogonal fusion module shown in Fig. 1 and directly splicing and fusing the multiscale feature $f_{s}$ with the global feature $f_{g}$. We also explored the fusion of two vectors using the Hadamard product, which is a common method for fusing two detectors. Table 5 lists the experimental results. Compared to the common fusion method of tensor splicing, our proposed method improves Recall@1 by 2.31% and 5.54% for Pitts30k and MSLS, respectively. This shows that through the process of orthogonal projection, redundant information in the multiscale features can be eliminated so that the output multiscale information is richer and more informative. Thus, a large number of shallow features in multiscale information does not affect the performance of global descriptors, thereby achieving complementary enhancement.

**Table 5:** Compare with other fusion strategies. Significant values are in bold.

Method	Pitts30k			MSLS		
	R@1	R@5	R@10	R@1	R@5	R@10
Hadamard	85.47	91.52	96.39	75.68	81.89	85.92
Concatenation	89.34	96.61	98.21	80.95	87.70	89.46
Orthogonal	**91.65**	**97.41**	**98.45**	**86.49**	**92.30**	**93.78**

Bold indicates optimal results.

## Conclusion

In this study, we proposed ConvMLP, a new feature-aggregation method for VPR that aggregates channel information through convolution and spatial information through adaptive average pooling. Experiments showed that this method can effectively deal with viewpoint changes, illumination changes, and appearance differences in VPR. Second, we proposed an orthogonal projection fusion multiscale feature extraction strategy for the problem in which traditional methods do not fully utilize the multiscale information generated by feature extraction and the information redundancy problem of traditional feature fusion methods. Our proposed framework eliminates as much redundant information as possible in multiscale features by spatial attention and orthogonal projection. The proposed architecture achieved the best Recall@1 of 91.65% and 86.49% on Pitts30k and MSLS, respectively, indicating that it can effectively avoid the problems of information underutilization and redundant feature fusion. Our proposed method achieved good performance on several publicly available VPR benchmark datasets, with improvements ranging from 0.1 to 5% over existing VPR methods, and outperformed the best existing methods by 5% on the most challenging Nordland dataset. The proposed method can be generalized to other image-retrieval tasks in addition to VPR.

However, this study has some limitations and areas for improvement. In this study, the performance saturation phenomenon occurred prematurely when ablation experiments were conducted with stacked ConvMLP quantities. In addition, the performance of our method can still be improved for some datasets and the proposed method is also difficult to apply in regions without reference image database. In the future, we will incorporate local feature matching to refine the global retrieval results and further improve VPR performance.

## Data Availability

The data analyzed during the current study are available in GSV-Cities at 10.1016/j.neucom.2022.09.127.
